# Beyond Food Preservation: Translational Perspectives of Synthetic Antioxidants

**DOI:** 10.3390/antiox15080973

**Published:** 2026-08-05

**Authors:** Daniela Vergara, Felipe Lizama, Carlos Arias-Fuentes, Emigdio Chávez-Ángel, Alejandro Castro-Alvarez

**Affiliations:** 1 Center of Excellence in Translational Medicine—Scientific Technological Bioresource Nucleus (CEMT-BIOREN), Laboratory of Pharmaceutical and Cosmetic Bioproducts, Faculty of Medicine, Universidad de La Frontera, Temuco 4811230, Chile; daniela.vergara@ufrontera.cl; 2 Millennium Nucleus Bioproducts, Genomics and Environmental Microbiology (BioGEM), Avenida España 1680, Valparaíso 2390123, Chile; 3 Biotechnology Undergraduate Program, Faculty of Agricultural Science and the Environment, Universidad de La Frontera, Temuco 4811230, Chile; f.lizama03@ufromail.cl; 4 Chemistry and Pharmacy Undergraduate Program, Faculty of Medicine, Universidad de La Frontera, Temuco 4811230, Chile; c.arias18@ufromail.cl; 5 Catalan Institute of Nanoscience and Nanotechnology, CSIC and BIST, Campus UAB, Bellaterra, 08193 Barcelona, Spain; 6 Departamento de Ciencias Preclínicas, Facultad de Medicina, Universidad de La Frontera, Temuco 4780000, Chile

**Keywords:** synthetic antioxidants, bioavailability, toxicity, neuroprotection, oncology, nanomedicine, translational medicine

## Abstract

Synthetic antioxidants are attracting more interest as research moves beyond their traditional use as preservatives in food and materials. Recent studies are exploring how these redox-active molecules might be used in medicine. This review looks at how synthetic antioxidants are made, what is known about their biological effects, and the challenges of using them for neuroprotection, cancer treatment, organ protection, and regenerative medicine. In this review, synthetic antioxidants are defined as synthetic or semisynthetic small molecules with redox activity. Other antioxidant systems, such as non-phenolic molecules, prodrugs, peptides, metal complexes, nanocarriers, nanozymes, and biomaterials, are mentioned as related but are not considered classic synthetic antioxidants. Some of these compounds and delivery systems have shown promising results in cell, tissue, and animal studies. They have been found to influence neuroinflammation, mitochondrial function, and tumor redox responses, and to protect tissues from ischemic or inflammatory damage. However, most of these candidates are still in preclinical development. Progress is slowed by issues such as inconsistent bioavailability, dose-dependent toxicity, unclear mechanisms of action, a lack of reliable biomarkers, and manufacturing or regulatory challenges, especially for advanced delivery systems. New research highlights the potential of hybrid molecules, organelle-targeted delivery, and systems that respond to specific triggers. Still, there is not enough strong clinical data to support these approaches. Moving forward, the field will need thorough pharmacological studies, careful safety checks, standardized ways to measure effectiveness, and well-designed clinical trials.

## 1. Introduction

The use of synthetic phenolic antioxidants (SPAs), such as butylated hydroxytoluene (BHT) and butylated hydroxyanisole (BHA), was primarily limited to the stabilization of materials and the food industry, where they acted as antioxidants by inhibiting lipid peroxidation [1,2]. However, the current understanding of oxidative stress as a causal factor contributing to various complex human diseases has spurred a shift from simple preservatives to rationally designed therapeutic agents [3,4]. This “renaissance” is based on strategic structural modifications designed to overcome the limitations of their natural counterparts, enabling the creation of molecules with optimized pharmacokinetics, greater chemical stability, and a superior ability to penetrate critical biological barriers, such as the blood–brain barrier [5]. A key aspect of this rational redesign is the improvement in metabolic stability. Unlike naturally occurring polyphenols, which are typically high-affinity substrates for phase II metabolic enzymes (primarily UDP-glucuronosyltransferases and sulfotransferases) [6,7], synthetic antioxidants incorporate specific structural modifications, such as the introduction of blocking functional groups at key phenolic or aromatic positions. Contemporary medicinal chemistry strategies focus on improving specific physicochemical and pharmacokinetic properties of antioxidant candidates, including chemical stability, metabolic resistance, and the capacity to penetrate the central nervous system. In certain compounds, modification of steric or electronic features at phenolic or aromatic positions can reduce susceptibility to phase II conjugation, potentially influencing clearance rates, plasma half-life, and tissue distribution. Nevertheless, these outcomes are compound-dependent and require authentication through pharmacokinetic studies. Structural modifications alone do not ensure increased bioavailability or improved tissue exposure.

Despite the vast array of compounds that have been developed, the “translational gap” remains a significant clinical challenge, hindering the adoption of these innovations into routine medical practice [8]; although numerous synthetic and semisynthetic antioxidants demonstrate robust efficacy in in vivo models and in vitro assays, their transition to clinical use is often disappointing [9]. This is often due to poor oral bioavailability, narrow therapeutic indices, or toxicities that do not manifest in the preclinical phases. Currently, only a few compounds, such as edaravone, have achieved regulatory approval for specific indications such as stroke or amyotrophic lateral sclerosis, underscoring the need for a paradigm shift in evaluation methodologies that more accurately account for the molecular context and subcellular distribution of drugs [10].

In this new therapeutic landscape, the development of mitochondria-targeted antioxidants (MTAs) represents a promising strategy for addressing cognitive decline and neurodegenerative diseases. Given that mitochondrial dysfunction and the accumulation of reactive oxygen species (ROS) are early and central events in the pathogenesis of Alzheimer’s disease [11,12], the development of molecules such as MitoQ, SkQ1, and MitoTEMPO has made it possible to directly intervene at the primary source of cellular oxidative damage [13]. Unlike natural vitamins, whose efficacy in human trials has been inconsistent, these synthetic antioxidants selectively accumulate in the mitochondrial membrane, successfully mitigating neuroinflammation and preserving synaptic plasticity in animal models, positioning them as key candidates for future clinical interventions.

**Figure 1:**
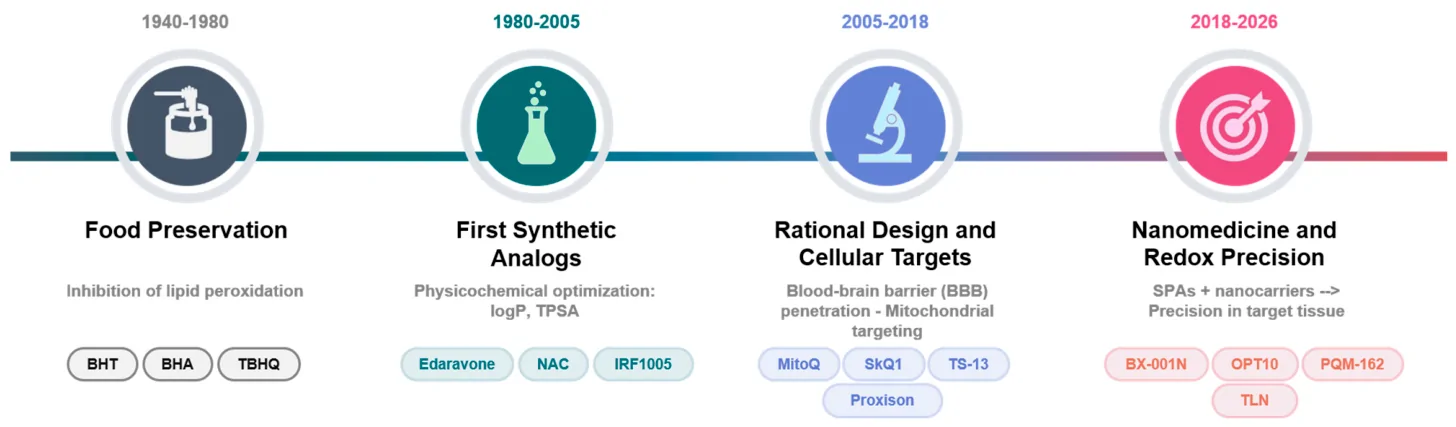
Historical evolution of synthetic antioxidants from food preservation agents to precision therapeutic tools, spanning four developmental eras (1940–2026). Nodes indicate representative compounds at each stage. The lower panel highlights the three therapeutic domains covered in this review: neuroprotection, oncology, and regenerative medicine.

The versatility of modern synthetic antioxidants extends to the modulation of regenerative and oncological processes through angiogenesis control. ROS act as critical regulators of angiogenic homeostasis, where low levels promote the neovascularization necessary for tissue healing, while excessive oxidative stress can trigger pathological angiogenesis linked to tumor growth or degenerative eye diseases [14]. In addition to classical synthetic antioxidants, this review examines related synthetic and semisynthetic antioxidant systems, such as N-acetylcysteine-based derivatives and curcumin analogs [15]. These compounds offer different ways to influence redox-sensitive pathways, including VEGF and HIF-1. We analyze them separately from classical synthetic antioxidants and evaluate their effects based on their molecular structure, the disease model used, dosage, route of administration, and the strength of the experimental evidence [16] (Figure 1).

## 2. Neuroprotection and Neurodegenerative Diseases

The success of synthetic antioxidants in neuroprotection depends on their ability to cross the blood–brain barrier (BBB) and reach specific organelles in the brain. Rational design strategies have led to the development of molecules such as Proxison, a synthetic flavonoid whose structure incorporates a lipophilic tail that facilitates diffusion across the cell membrane and moderate mitochondrial accumulation. As reported by Drummond et al., this modification appears to enhance potency in comparison to natural flavonoids in protecting against acute oxidative stress in the specific models tested [17]. Similarly, Khrapova et al. have demonstrated that monophenolic compounds such as TS-13 possess optimized lipophilicity that allows them to penetrate the BBB following systemic administration, reaching therapeutically significant concentrations in brain tissue [18].

This penetration capacity is not solely due to a general increase in lipophilicity, but rather to a deliberate optimization of the physicochemical descriptors that govern passive transport across the cerebral endothelium. As summarized in Table 1, the controlled reduction of hydrogen-bond donors (HBDs) drastically decreases the desolvation energy required for the molecule to leave the aqueous plasma medium and integrate into the lipid bilayer of the blood–brain barrier. While natural flavonoids and polyphenols possess multiple hydroxyl groups that increase their HBD and polar surface area (PSA > 100 Å^2^), severely limiting their transcellular diffusion, compounds such as TS-13 or Proxison have been designed to maintain an octanol/water partition coefficient (logP) within the optimal range for the CNS (~2.0–~5.5) and a PSA of less than 100 Å^2^, thereby ensuring efficient brain permeability without compromising their redox activity. This rational design strategy, supported by the comparative parameters presented below, overcomes one of the main historical pharmacokinetic limitations of botanically derived antioxidants.

In this same vein of structural optimization, the development of synthetic derivatives of natural phenolic acids has gained prominence. As noted by Nabavi et al., the synthesis of gallic acid derivatives aims to improve bioavailability and in vivo antioxidant potency compared to the natural compound, demonstrating superior efficacy in modulating systemic oxidative stress and in models of post-stroke depression [20].

Complementing these structural advances, the rational engineering of nanoplatforms now represents a cornerstone strategy for blood–brain barrier permeation. Systems such as PLGA nanoparticles, PEGylated liposomes, and functionalized inorganic carriers (e.g., LMC-RES) markedly improve brain accumulation and sustain pharmacological release, translating into reduced Aβ deposition and oxidative stress in transgenic models [21,22]. Coupled with prodrug approaches and surface conjugation to BBB-specific ligands (e.g., anti-transferrin receptor antibodies, RVG29 peptide), these platforms enable receptor-mediated transcytosis that bypasses the passive diffusion limitations inherent to conventional natural antioxidants [23].

Building on these delivery strategies, the development of synthetic catechols has yielded promising results. Nuñez-Figueredo et al. described KM-34, a rationally designed molecule that exhibits potent neuroprotective activity against oxidative agents. This compound stands out for its ability to prevent lipid peroxidation and mitochondrial dysfunction by acting through iron coordination and the direct scavenging of free radicals, positioning it as an effective candidate for mitigating neuronal damage in various degenerative diseases [24].

In the context of Parkinson’s disease, the development of synthetic isothiocyanates, such as ITC-57, studied by Lee et al., demonstrated that neuroprotection can be achieved through a dual mechanism: potent direct antioxidant activity and a robust anti-inflammatory response mediated by the Nrf2/ARE pathway. This compound not only reduces the death of dopaminergic neurons but also attenuates microglial activation [25]. Complementarily, the synthetic chalcone AN07, described by Chen et al., has exhibited similar properties by protecting neurons against MPP+-induced neurotoxicity through inhibition of the NF-κB pathway and reduction in endoplasmic reticulum stress [26].

The fight against Alzheimer’s disease has benefited from the development of synthetic hybrids designed to target multiple targets. The compound evaluated by Xie et al., a derivative of 8-hydroxyquinoline and resveratrol, has shown a remarkable ability to inhibit β-amyloid (Aβ) peptide aggregation and chelate transition metals, thereby reducing plaque formation and oxidative damage [27]. Furthermore, the work by Nawaz et al. on synthetic progesterone derivatives has emerged as a promising strategy for restoring cholinergic function and reducing oxidative stress, demonstrating effective inhibition of cholinesterase enzymes along with normalization of glutathione and superoxide dismutase levels [28].

**Figure 2:**
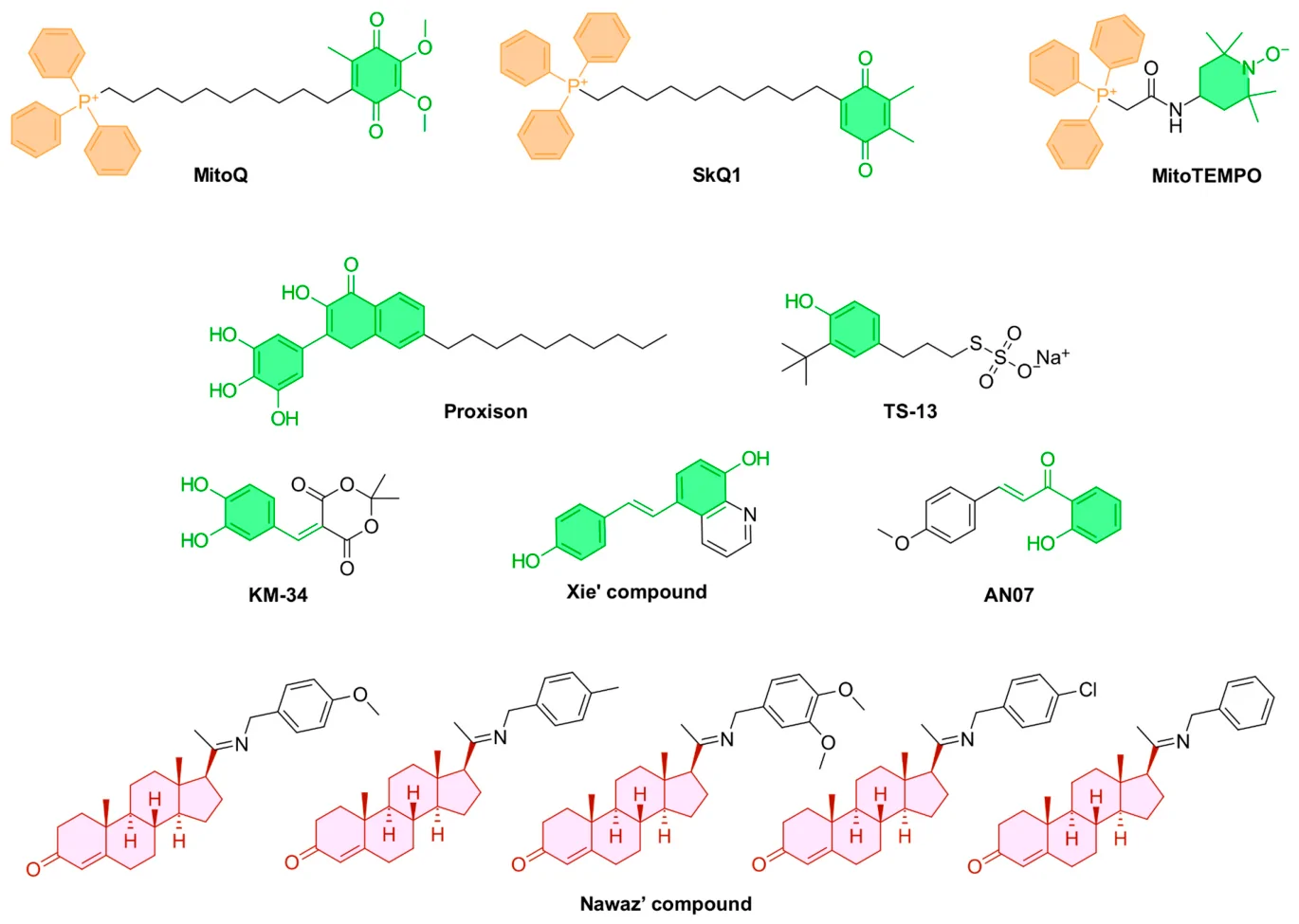
2D images of the structures mentioned, with their potential therapeutic uses listed below each name. The orange section represents the region responsible for mitochondrial uptake, the green section corresponds to the regions that confer antioxidant activity, and the pink structures represent progesterone analogs.

One of the most significant challenges in optimizing these compounds lies in distinguishing between direct free radical neutralization and modulation of cellular signaling pathways. While results from Duong et al. suggest that molecules such as t-butyl bisphenol act primarily through direct scavenging [29], compounds like AN07 and ITC-57 function as “indirect antioxidants” by regulating protective protein expression and suppressing pro-inflammatory cascades [25,26]. KM-34 (Figure 2) exemplifies a convergent strategy: despite its high direct scavenging and iron-chelation capacity, its neuroprotective effect ultimately preserves mitochondrial integrity, suggesting a systemic impact on cell viability under persistent oxidative stress [24].

Nevertheless, robust preclinical efficacy has not systematically translated into clinical success because of critical translational bottlenecks. Interspecies variability in CNS pharmacokinetics and BBB transporter expression complicates dose extrapolation from animal models to humans [30]. Moreover, the absence of validated cerebrospinal fluid biomarkers for mitochondrial oxidative stress (e.g., F2-isoprostanes, carbonylated tau, 4-HNE) hinders patient stratification and obscures the correlation between brain exposure and therapeutic response [31]. These gaps necessitate a shift toward adaptive clinical trial designs with redox-stratified cohorts and standardized mechanistic endpoints before synthetic antioxidants can advance beyond phase II [32].

## 3. Oncology: Chemosensitization and Protection of Healthy Tissue

The use of antioxidants in oncology presents a therapeutic paradox that falls under the concept of the “Redox Paradox.” In this context, tumor cells exhibit elevated levels of ROS, which can play a dual role: on the one hand, promoting pro-tumor signaling (favoring cell survival and proliferation) and, on the other, inducing anti-tumor signaling through the generation of oxidative stress [33].

This apparent contradiction is explained by the sustained activation of antioxidant systems in tumor cells, where the Nrf2 transcription factor of the Keap1/Nrf2/ARE axis plays a central role by inducing the expression of genes involved in glutathione (GSH) synthesis, detoxification of xenobiotics, and elimination of ROS, thereby regulating cellular redox homeostasis. This allows cancer cells to tolerate elevated levels of oxidative stress, promoting the formation of a functional antioxidant shield and increasing intracellular redox buffering capacity, which is associated with a higher concentration of reducing agents that buffer oxidative fluctuations [34].

In contrast, under normal physiological conditions, Nrf2 is strictly regulated through its interaction with Keap1, which limits its basal activity. Its persistent activation in tumor cells can confer an adaptive advantage, contributing to resistance to anticancer therapies. This dependence on an adaptive redox state presents pharmacological interest as a therapeutic target, since altering this balance by exceeding the redox buffering capacity of tumor cells can induce lethal oxidative stress, reflecting a dose-dependent hormetic-type response. Although reducing oxidative stress could, theoretically, protect tumor cells from ROS-induced apoptosis, the selective mitigation of oxidative damage in healthy tissues is essential to prevent chemotherapy-induced systemic toxicity [35].

Changes in glucose metabolism and pentose phosphate pathway activity can help many tumors produce NADPH, but how much this happens and how important it is can vary between cancer types and genotypes [36]. So far, there is no strong evidence that synthetic antioxidants as a group block glycolysis, pentose phosphate pathway activity, or nucleotide production. Instead, certain synthetic antioxidants, hybrid molecules, or redox-responsive delivery systems might affect redox buffering, metabolic enzymes, or how treatments work in specific experimental models. These effects should be linked to the specific compound and model used, and any suggested mechanisms should be clearly distinguished from those that have been directly proven [37].

In this scenario, synthetic antioxidants emerge as dual agents capable of differentially modulating redox responses. Recent evidence suggests that certain synthetic antioxidants not only do not interfere with the cytotoxic efficacy of chemotherapeutic agents but may also enhance their oncolytic activity while safeguarding vital organs, such as the heart and liver. Researchers tested TS-13 with doxorubicin in a mouse model of Lewis lung carcinoma. Tumor growth inhibition was 55.4% for the combination and 49.5% for doxorubicin alone [38]. This difference should not be seen as proof of potentiation unless the original study includes a direct statistical comparison, an effect size, and sufficient sample-size information. Still, these results show that TS-13 did not reduce doxorubicin’s antitumor effect under the tested conditions and suggest that more research on TS-13 is needed.

**Figure 3:**
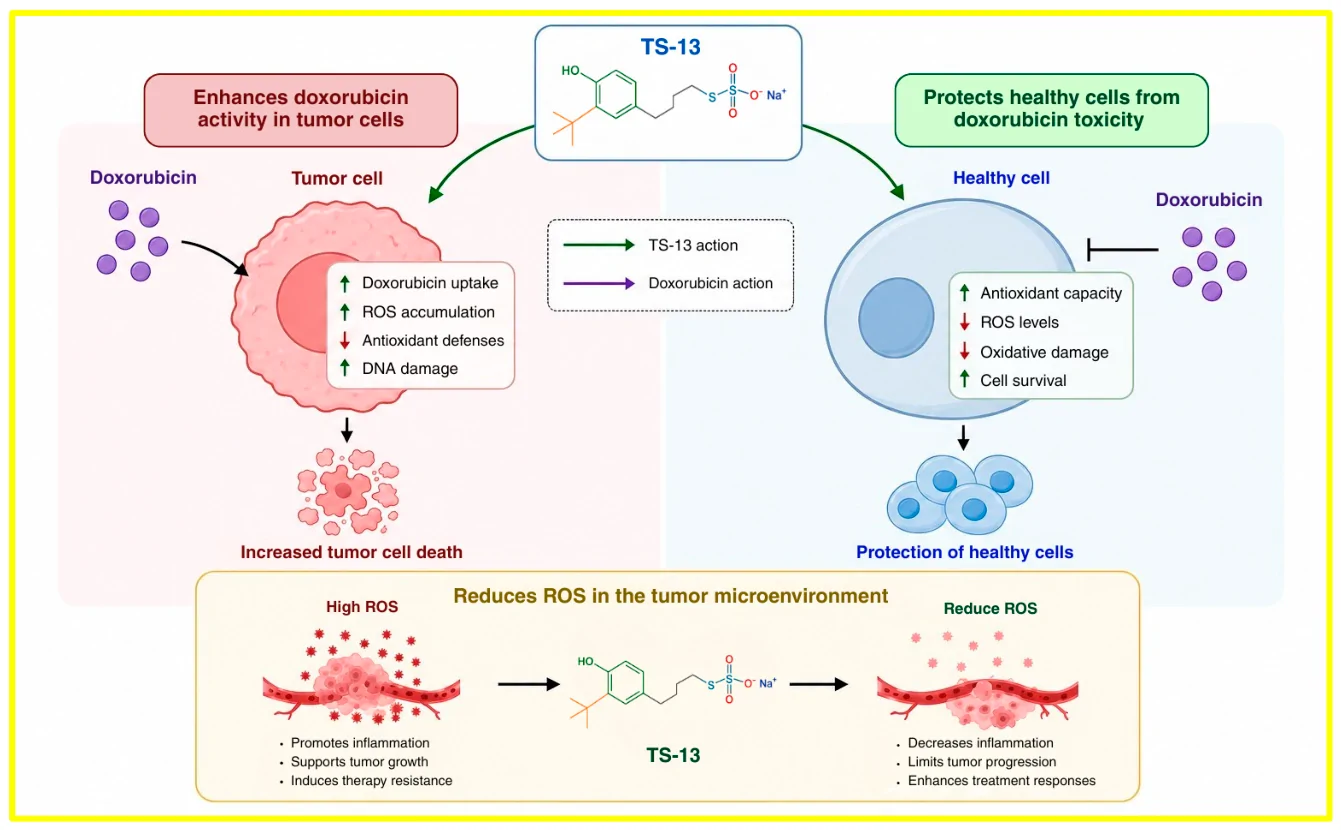
The impact of TS-13 on its dual action of enhancing doxorubicin activity and protecting healthy cells, as well as reducing ROS in the tumor microenvironment.

The ability of synthetic antioxidants to protect healthy tissues against chemotherapy-induced toxicity is equally promising and linked to the activation of endogenous cellular defense pathways. In models of doxorubicin-induced cardiotoxicity, administration of TS-13 not only reduced the cytostatic’s cachectic effect but also significantly improved the functional activity of isolated hearts, increasing coronary flow, ventricular pressure, and myocardial contractility [39]. This cardioprotective effect is attributed to TS-13’s ability to act as an activator of the redox-sensitive Keap1/Nrf2/ARE signaling system, the primary regulator of redox homeostasis, thereby protecting cardiomyocytes without negating the antitumor activity of the drug (Figure 3). Similarly, rationally designed synthetic hybrids by Baptistella et al., such as the resveratrol-curcumin derivative PQM-162, have shown chemopreventive effects in pre-neoplastic colon lesions, reducing inflammation (decreased TNF-α and COX-2) and oxidative stress (increased Nrf2) without apparent systemic toxicity [40]. These findings reinforce the feasibility of using synthetic antioxidants to improve the therapeutic index of cancer treatment.

In addition to tissue protection, modern synthetic antioxidant designs aim to directly target tumor resistance through specific antioxidant mechanisms. Recent hybrid compounds, such as the oxadiazole-chalcone hybrids developed by Patil et al. (e.g., compound 97A), have demonstrated potent inhibitory activity against EGFR-resistant lung cancer cell lines (L858R/T790M mutation), combining selective cytotoxicity with robust antioxidant activity assessed in DPPH and ABTS assays [41]. Along the same lines of structural optimization, Kannan et al. reported the synthesis of a semisynthetic curcumin analog through the incorporation of a benzothiazole ring, which significantly enhanced its oncolytic efficacy [42]. This derivative not only exhibits high antioxidant capacity (ABTS) but also demonstrates potent and selective cytotoxicity against various cell lines, including lung cancer (A549), cervical cancer (HeLa), and breast cancer (MDA-MB-231). Its safety profile has been validated through in vivo acute toxicity studies, demonstrating robust antitumor effects in preclinical models, suggesting a safety profile that warrants further investigation regarding the viability of the host organism [42].

Similarly, Hou et al. reported the efficacy of synthetic soliquiritigenin (S-ISL), which was shown to inhibit the proliferation and metastasis of squamous cell carcinoma of the tongue by reducing intracellular ROS and modulating the Bax/Bcl-2 ratio, thereby inducing apoptosis without significantly affecting non-malignant cells [43]. Taken together, these studies suggest that the “antioxidant paradox” might be addressed through the rational design of synthetic molecules that exploit the metabolic differences between tumor and healthy cells, positioning synthetic antioxidants not only as protective agents but as integral components of chemosensitization strategies and precision medicine in oncology.

## 4. Organ Protection and Regenerative Medicine

Yan et al. reported that BX-001N preferentially accumulated in injured renal tissue and resulted in more favorable renal injury-associated endpoints compared to free bilirubin in the ischemia–reperfusion model [44]. The formulation was linked to reductions in oxidative and inflammatory markers as well as improvements in indices of tubular recovery. While these data support further preclinical evaluation, they do not establish clinical efficacy.

Nanocarriers can change how drugs are released and distributed in the body in comparison to traditional drug delivery. These changes depend on components such as what the carrier is made of, how much drug it holds, how quickly it breaks down, and how it is administered. The intended release pattern should only be reported if it has been clearly shown. Some nanocarrier systems might lower peak drug levels or change where drugs go in the body, which could help reduce certain side effects. Still, there is not much long-term safety data for these systems. Because of this, nanocarrier formulations should not be called safer or more effective than standard treatments unless there is explicit comparative data on how the body processes the drug and its safety.

Studies show that the TLN prodrug improves mitochondrial delivery to proximal tubular cells and yields better pharmacokinetic or protective outcomes than free NAC in the tested model [45]. PtS pre-nanozymes and poly(propylene sulfide) nanoparticles have also shown positive effects on tissue protection in preclinical renal and transplant models [46,47]. Still, these results are specific to the models used and do not prove clinical effectiveness or long-term safety.

Extending these organ-protective strategies to pancreatic transplantation, the success of pancreatic islet transplantation in the treatment of type 1 diabetes depends critically on cellular viability in the face of post-isolation hypoxic and oxidative stress. In this context, the use of synthetic bilirubin analogs, which avoid the use of animal-derived products, represents a milestone in clinical safety. Research by Luckring et al. confirms that these analogs possess dose-dependent cytoprotective activity, preserving the integrity of rat islets and reducing the release of damage-associated molecular patterns (DAMPs) [48]. Complementing this approach, thioketal-based synthetic antioxidants have demonstrated the ability to protect pancreatic islets against ROS-mediated injury. Raj et al. reported that thioketal treatment reduced oxidative damage, preserved islet cell function in vitro, and improved marginal islet mass engraftment in diabetic mice [49].

These antioxidant protection strategies now extend to tissue engineering through redox-active biomaterials. Hydrogels such as CPP-L/GelMA, which integrate catalase and oxygen-generative nanoparticles, reverse local hypoxic oxidative stress, activating the Nrf2-BMAL1-autophagy axis to enhance angiogenesis and bone regeneration [50]. Similarly, Gel/ZIFC composite scaffolds, based on nitrogen-doped carbon nanozymes, accelerate bone repair by activating endogenous antioxidant defenses while maintaining structural conductivity [51]. These systems illustrate how synthetic antioxidants and their programmable vehicles are redefining regenerative medicine beyond simple cell transportation.

The integrity of genetic material in germ cells is a cornerstone of reproductive medicine, frequently threatened by ROS-induced sperm DNA fragmentation. The design of Schiff base complexes derived from L-asparagine and heavy metals has emerged as a sophisticated chemical strategy to mitigate this damage. The work by Suliman et al. highlights that these ligands and their metal complexes act as potent free radical scavengers, demonstrating in comet assays a superior ability to maintain DNA integrity under conditions of severe oxidative stress [52]. This application of coordination chemistry enables the creation of protective agents with optimized thermal and chemical stability for complex biological environments.

**Figure 4:**
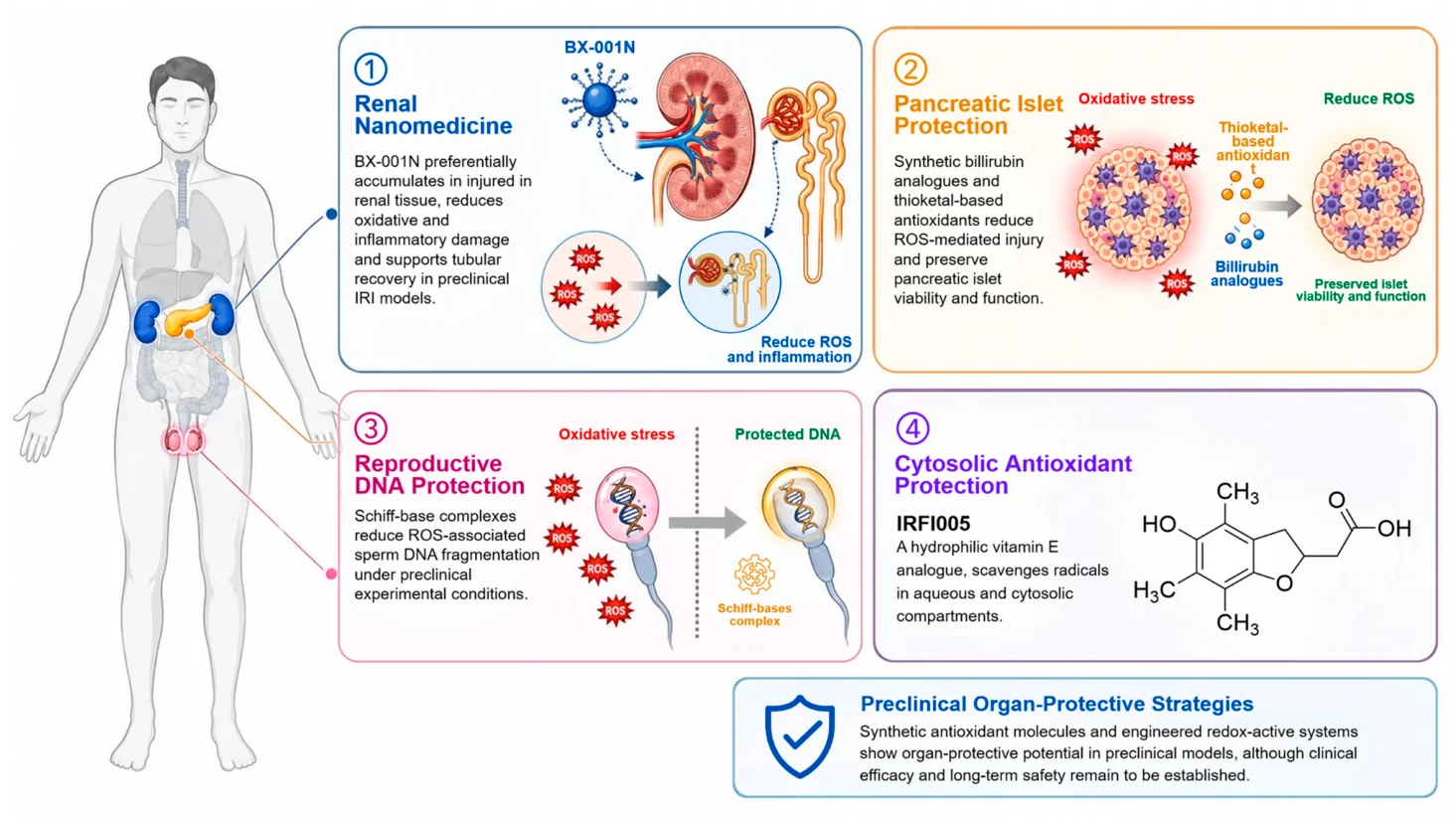
The scheme illustrates several engineered antioxidant interventions studied for tissue preservation and regenerative medicine. It includes a bilirubin-based renal nanomedicine, strategies to protect pancreatic islets, Schiff-base complexes tested for sperm DNA protection, and the hydrophilic vitamin E analog IRFI005. These treatments belong to different therapeutic categories and do not share the same mechanisms, evidence levels, or clinical development stages. Most of the available data come from in vitro and animal studies.

For this reason, the transition from natural lipophilic antioxidants to synthetic analogs with improved pharmacokinetics is essential for systemic organ protection. The study by Barzegar et al. on IRFI005, a hydrophilic analog of vitamin E, illustrates how molecular design can facilitate free permeation through cell membranes [53]. Unlike natural α-tocopherol, this compound allows for efficient radical neutralization in cytosolic compartments, protecting cells against hydroperoxide-mediated toxicity (Figure 4). The combination of semi-empirical calculations and biological assays confirms that optimizing the electron- and proton-donating capacity is key to maximizing the reducing power of these agents in translational medicine [53].

This transition toward high-performance systemic analogs is reflected in hepatic and cardiac protection, where subcellular targeting is critical. In hepatic ischemia–reperfusion injury, the polymeric conjugate OPT10 (TEMPO-functionalized) outperforms NAC and GSH by scavenging mitochondrial ROS and promoting anti-inflammatory macrophage polarization [54], while nanozymes like TBP@CeO restore mitochondrial respiratory chain function in acetaminophen-induced toxicity [55]. In cardiac transplantation, chitosan nanoparticles loaded with CoQ10 and modified with the SS31 peptide (CoQ10@TNPs) protect cardiac grafts during cold storage, mitigating mitochondrial oxidative stress and improving post-transplant function [56]. Furthermore, hybrid platforms such as VB@MOF/TA and D/Ce@mPDA-C/P combine iron chelation, hierarchical ROS scavenging, and ferroptosis modulation, demonstrating that the synergy between synthetic antioxidants and smart nanovehicles is key to overcoming the narrow therapeutic index of natural agents [57,58].

## 5. The Translational Gap

The transition of synthetic antioxidants from the design stage to commercialization represents one of the most complex challenges in modern pharmacology (see Table 2). Natural antioxidant treatments have demonstrated variable success in clinical settings. This variation is ascribed to factors including low bioavailability, unstable metabolic profiles, limited target engagement, heterogeneity in patient selection, and the absence of reliable pharmacodynamic biomarkers. The capacity of a compound to scavenge free radicals in cell-free assays does not necessarily predict its potency in vivo. Comparisons between natural and synthetic compounds should be carried out using identical models, dosages, and exposure conditions as those employed in the original research studies. Although structural modification of a compound may enhance certain properties, it can also bring about new challenges related to distribution, metabolism, or toxicity [17]. The advantage of synthetics lies in the engineering of their lipophilicity; for example, the addition of hydrocarbon tails allows molecules such as TS-13, analyzed by Khrapova et al., to cross the blood–brain barrier and reach therapeutic concentrations in the brain parenchyma [18]. However, this “advantage” is often the root of the translational problem: optimization for cellular penetration does not always guarantee predictable pharmacokinetics in humans, a point that Squillace et al. identify as a critical gap in the treatment of cognitive impairment, where in vivo evidence rarely translates to clinical success [59].

There is a fundamental, yet often overlooked, distinction between the doses of synthetic antioxidants used as food preservatives (traditional synthetic antioxidants such as BHT or BHA) and the pharmacological doses required to treat conditions such as ischemia or cancer. Tauchen et al. emphasize that the state of the art is stalled at this transition; compounds that are safe at trace levels to prevent lipid rancidity may exhibit unacceptable toxicity profiles when administered at high doses [9]. The gap widens when preclinical studies fail to model long-term accumulation, leading to unexpected failures in Phase I and II safety trials.

One of the most profound debates in translational medicine is whether the benefits of synthetic antioxidants stem from their direct scavenging action or from their ability to modulate intracellular signaling pathways. The evidence suggests that simply neutralizing ROS is insufficient. According to Lee et al., the success of molecules such as ITC-57 lies not only in their antioxidant capacity but also in their role as indirect modulators via the Nrf2/ARE pathway, which activates a much more robust and long-lasting endogenous cytoprotective response [25]. Similarly, Patil et al. demonstrate that in oncology, the efficacy of synthetic hybrids depends on their ability to inhibit specific targets such as EGFR, transforming the antioxidant into a selective modulator rather than a “sponge” for free radicals [41]. This mechanistic complexity complicates the standardization of assays, as measuring “antioxidant power” in blood does not necessarily reflect genomic modulation in the target tissue.

Finally, regulatory and social factors act as an insurmountable bottleneck. As Tauchen et al. discuss, there is a persistent stigma against “synthetic” substances in the approval of drugs aimed at wellness and prevention, often favoring “natural” compounds that, although less effective, are perceived as safer [9]. This perception forces synthetic antioxidants to undergo much more rigorous toxicity testing than their natural counterparts. Furthermore, Squillace et al. emphasize that the lack of precise biomarkers to measure mitochondrial oxidative stress in humans prevents regulatory agencies from validating the efficacy of new compounds targeting specific organelles, leaving promising molecules in a permanent preclinical “limbo”.

## 6. Future Directions and Conclusions

The future of translational medicinal chemistry is moving away from the concept of antioxidants as mere radical scavengers and toward the design of smart, multifunctional agents. A major trend is the development of hybrid molecules that combine an antioxidant core with targeted drug carriers, such as those that act on EGFR mutations or the Wnt/β-catenin pathway, to control the complex interactions between inflammation and oxidative stress in cancer and neurodegeneration. In addition, the design of smart synthetic antioxidants to be activated by specific stimuli inherent to the microenvironment, such as tumor acidity or critical levels of reactive oxygen species (ROS), maximizes therapeutic impact while minimizing unwanted systemic effects.

Furthermore, substantial progress in this area will depend on the continuous refinement of nanocarriers for targeted delivery. The conjugation of synthetic analogs with nanoparticle systems addresses solubility challenges and ensures concentrated accumulation in damaged organs. This approach is proving vital for regenerative medicine and reproductive health, where coordination complexes and nanoplatforms protect sensitive payloads from degradation, maintaining their integrity in complex biological environments.

Moreover, the literature highlights a therapeutic renaissance of synthetic antioxidants. There is a clear need for the scientific community to distinguish these rationally designed compounds from natural nutritional supplements, as their structural versatility offers a unique pharmacological platform. Looking ahead, the focus must shift from basic radical neutralization to the modulation of signaling pathways—such as the Nrf2 axis—and metabolic regulation. Clinical success is likely to be achieved by integrating these two mechanisms: providing robust cytoprotection while simultaneously offering a synergistic therapeutic intervention.

Finally, realizing this potential requires addressing critical unresolved barriers, including the lack of validated oxidative stress biomarkers, the need for more extensive clinical trials, challenges in the reproducibility of nanomedicine manufacturing, and the establishment of standardized mechanistic endpoints.

Overall, and despite these unresolved barriers, the balance remains favorable. The disadvantages of synthetic antioxidants are dose-, design-, and manufacturing-dependent, and are therefore amenable to optimization, whereas their advantages, metabolic stability, tunable biodistribution, access to the central nervous system, defined and reproducible composition, and the capacity to act as targeted modulators of endogenous cytoprotective signaling, address precisely the limitations that have historically prevented natural antioxidants from succeeding in the clinic. Provided that these molecules are evaluated according to conventional pharmacological standards rather than the expectations applied to dietary supplements, the prospective clinical benefit of rationally designed synthetic antioxidants outweighs their possible drawbacks.

## Figures and Tables

**Table 1:** Comparative physicochemical profiles of selected synthetic antioxidants and representative natural polyphenols. The molecular descriptors governing central nervous system permeability and oral bioavailability are reported: partition coefficient (logP), topological polar surface area (TPSA, Å^2^), number of hydrogen bond donors (HBD), and hydrogen bond acceptors (HBA). Values were calculated using SwissADME [19].

Compound	logP	TPSA (Å^2^)	HBD	HBA	BBB Permeability	Origin/Class
TS-13	~2.27	~97.28	1	4	High	Synthetic (Monophenol)
Proxison	~5.37	~97.99	4	5	High-Moderated	Synthetic (Modified flavonoid)
Gallic acid	~0.21	~97.99	4	5	Very low	Natural (Polyphenol)
Quercetin	~1.23	~131.36	5	7	Low	Natural (Flavonol)
Kaempferol	~1.20	~111.13	4	6	Low	Natural (Flavonol)
Epigallocatechin gallate	~0.95	~197.37	8	11	Very low	Natural (Catequin)

**Table 2:** Synthetic antioxidants discussed in the review: compound, chemical class, molecular target, experimental model, reported efficacy, and translational status.

Comp.	Chemical Class	Molecular Target	Experimental Model	Reported Efficacy	Translational Status	Clinical Area
**TS-13**	Synthetic monophenol	Keap1/Nrf2/ARE, Mitochondria	Lewis LC (murine), cardiomyocyte (ex vivo)	↓55.4% tumor; ↑coronary flow	Advanced preclinical	Oncology/Cardio
**Proxison**	Synthetic flavonoid	Cell membrane, Mitochondria	Neuronal (acute oxidative stress)	Potency > natural flavonoids	Preclinical	Neuroprotection
**KM-34**	Synthetic catechol	Fe^2+^ chelation, direct ROS	Neurotoxicity in vitro/in vivo	↓lipid peroxidation, ↑neuronal viability	Preclinical	Neuroprotection
**ITC-57**	Synthetic isothiocyanate	Nrf2/ARE, microglia (anti-inflam.)	MPTP (murine Parkinson’s)	↓dopaminergic death, ↓microglial activation	Preclinical	Neuroprotection (PD)
**AN07**	Synthetic chalcone	NF-κB, ER stress	MPP^+^ neurotoxicity	Neuroprotection vs MPP^+^ in vitro	Preclinical	Neuroprotection (PD)
**PQM-162**	Resv-Curcumin hybrid	Wnt/β-catenin, Nrf2, COX-2	Preneoplastic colon lesions (murine)	↓TNF-α, ↓COX-2, ↑Nrf2	Preclinical	Oncology (chemoprev.)
**97A**	Oxadiazole-chalcone hybrid	EGFR (L858R/T790M), ROS	Resistant NSCLC lines	Selective IC_50_ in mutant EGFR	Preclinical	Oncology (lung)
**S-ISL**	Synthetic isoliquiritigenin	Bax/Bcl-2, intracellular ROS	Tongue squamous carcinoma	↓proliferation/metastasis	Preclinical	Oncology
**BX-001N**	Bilirubin-PEG nanopart.	Renal ROS, inflammatory cells	Renal IRI (murine)	↑tubular regeneration, ↓infiltrate	Preclinical	Regenerative Medicine
**OPT10**	TEMPO-polymer (conjugate)	Mitochondrial ROS, M2 macrophage	Hepatic IRI	Outperforms NAC and GSH in IRI	Preclinical	Regenerative Medicine
**TLN**	TPP^+^-chitosan-NAC prodrug	Tubular mitochondria (megalin)	Acute renal IRI	↑PK vs free NAC	Preclinical	Regenerative Medicine
**Edaravone**	Synthetic pyrazolone	ROS (direct)	Ischemic stroke, ALS	↓neurological deficit (approved CT)	Approved (FDA/EMA)	Neuroprotection
**IRFI005**	Hydrophilic Vit. E analog	Peroxyl radical (cytosol)	Hydroperoxide toxicity	↑reducing activity vs α-tocopherol	Preclinical	Systemic protection
**GP13**	Synthetic peptide (Spirulina)	GLUT-4 (skeletal muscle)	L6 myoblasts (in vitro)	↑glucose uptake + antioxidant effect	Preclinical	Regenerative Medicine (DM1)

Abbreviations: PK = pharmacokinetics; IRI = ischemia–reperfusion injury; NSCLC = non-small cell lung cancer; DM1 = type 1 diabetes mellitus; ALS = amyotrophic lateral sclerosis; NAC = N-acetylcysteine; PD = Parkinson’s disease; ↓ = reduced; ↑ = increases.

## Data Availability

No new data were created or analyzed in this study. Data sharing is not applicable to this article.
